# New hepatitis C virus infection, re-infection and associated risk behaviour in male Irish prisoners: a cohort study, 2019

**DOI:** 10.1186/s13690-021-00623-2

**Published:** 2021-06-08

**Authors:** Des Crowley, Gordana Avramovic, Walter Cullen, Collette Farrell, Anne Halpin, Mary Keevans, Eamon Laird, Tina McHugh, Susan McKiernan, Sarah Jayne Miggin, Ross Murtagh, Eileen O. Connor, Marie O’Meara, Deirdre O. Reilly, John S. Lambert

**Affiliations:** 1 School of Medicine, University College Dublin, Dublin, Ireland; 2 Irish Prison Service, Dublin, Ireland; 3 Trinity College Dublin, Dublin, Ireland; 4 Department of Infectious Diseases, Mater Misericordiae University Hospital, Dublin, Ireland; 5 St. James’ Hospital, Dublin, Ireland; 6 Mater Misericordiae University Hospital, Dublin, Ireland; 7 Abbvie Ltd, Dublin, Ireland

**Keywords:** Hepatitis C, HCV, Prisoner, Prison, Incident, Harm reduction, Medication assisted treatment, MAT

## Abstract

**Background:**

Prisoners are recognised as a high-risk population and prisons as high-risk locations for the transmission of hepatitis c virus (HCV) infection. Injecting drug use (IDU) is the main driver of HCV infection in prisoners and harm reduction services are often suboptimal in prison settings. HCV prevalence and incident data in prisoners is incomplete which impacts the public health opportunity that incarceration provides in identifying, treating and preventing HCV infection. The aim of this study is to identify new HCV infection and associated risk factors in an Irish male prison.

**Methods:**

We conducted a follow up (18-month) cohort study on prisoners who had previously tested negative, self-cleared or had been successfully treated for HCV infection. We conducted the study in a male medium security prison located in Dublin Ireland (Mountjoy Prison) using HCV serology, a review of medical records and a researcher-administered questionnaire.

**Results:**

99 prisoners with a mean age of 33.2 yrs. participated in the study and 82(82.8%) completed a research-administered questionnaire. Over half (51%) had a history of drug use from a young age (14.8 yrs.), 49.9% a history of heroin use and 39% a history of IDU. The prevalence of HIV and hepatitis B virus core antibody was 3% and HCV antibody was 22.2%. No new HCV infections were identified in those who had never been infected (*n* = 77), had self-cleared (*n* = 9) or achieved sustained virological response (*n* = 12). Small numbers of prisoners continued to engage in risk-behaviour including, IDU both in the prison (*n* = 2) and the community (*n* = 3), sharing syringes (*n* = 1) and drug taking paraphernalia (*n* = 6) and receiving non-sterile tattoos (*n =* 3).

**Conclusion:**

Despite the high numbers of Irish prisoners with a history of IDU and HCV infection, new HCV infection is low or non-existent in this population. Small numbers of prisoners continue to engage in risk behaviour and larger studies are required to further understand HCV transmission in this cohort in an Irish and international context.

## Background

Hepatitis C (HCV) infection is a major public health concern and a leading cause of liver-related morbidity and mortality worldwide [1]. Injecting drug use (IDU) is the major driver of HCV infection in developed countries [1, 2]. Ongoing criminalisation of people who inject drugs (PWID) ensures an over representation of PWID and HCV infection in prison populations [3].

Epidemiological data on HCV infection in prisoners is lacking in many countries [3]. From available data it is estimated that over a quarter of prisoners globally have been infected with HCV increasing to over 60% in prisoners with a history of IDU [3]. Incident infection is estimated at 1.4 per 100 person-year (py) increasing to 16.6 per 100 py in prisoners with a history of IDU [3].

Ireland has one of the lowest incarceration rates in Europe with approximately 4000 people incarcerated across 14 locations in the Irish Prison Service (IPS) daily [4]. There are well-established medication assisted treatment (MAT) services across the entire prison estate but needle and syringe programmes (NSPs) are not available at any Irish prison location [5].

Studies on Irish prisoners report high rates of opioid use (50%), IDU (43%) and HCV infection (13–37%) [6–8]. Recent national HCV screening guidelines recommend the screening of all prisoners and re-screening annually with targeted screening if a HCV transmission risk is identified [9]. However, these national guidelines have yet to be fully implemented. HCV treatment in Irish prisons is provided by specialist services. Ireland, like other developed countries, has a large proportion of undiagnosed and untreated HCV-infected individuals incarcerated in its prisons [10].

The study site is one of three locations where in-reach hepatology services, through specialist nurses, are provided in the IPS. HCV direct-acting antivirals (DAAs) have been available in Ireland since 2014, with initial availability restricted on clinical need. DAAs (including 8-week and pan-genotypic regimens) can now be prescribed to all HCV-infected people needing treatment including prisoners.

This study is a follow up study from a previously published 2017 cross sectional study that estimated the prevalence of untreated chronic HCV infection and associated risk factors in a male Irish prisoners [11].

The 2017 study (*n* = 422) reported a HIV and hepatitis B virus (HBV) core antibody prevalence of 4.0 and 3.0% respectively and a HCV antibody prevalence of 22.8% among the general prison population, increasing to 79.7% among prisoners with history of IDU [11]. Of the HCV antibody positive prisoners, 11% were co-infected with HIV. It also reported a chronic untreated HCV prevalence of 13.1% [11]. Of those with chronic infection; 58.7% were infected with Genotype 1A and 41.3% with Genotype 3.

Similar to other national and international studies identified risk factors for HCV infection included: IDU, having received a prison tattoo and sharing syringes and drug taking paraphernalia [11–14].

A recent peer-led active case finding initiative at the study site found high levels of undiagnosed HCV infection and related liver disease (over 25% of those fibroscanned showed evidence of liver disease) [10]. Peer-supported screening identified 50 cases (12% of the study population) of active untreated HCV infection of which 19 (5% of the study population) had not been identified at committal. Of those identified 86% were linked with HCV care, with 33% undergoing or completing treatment.

Similar to other jurisdiction, Ireland struggles to increase rates of HCV screening and treatment in prisoners [12, 15, 16]. Barriers identified include: lack of knowledge, historical requirement to have a liver biopsy, the requirement to go to hospital, concerns regarding confidentiality, stigma experienced and inconsistent and delayed access to prison health services [17, 18].

Enablers identified include; access to health care, opt-out screening at committal, peer support, stability of prison life and in-reach hepatology services and fibroscanning [17].

The aims of this study are to estimate the HCV incident infection among Irish male prisoners and to describe levels of risk behaviour among prisoner who are aware of not having HCV infection or having a documented sustained virological response (SVR) post-treatment. This study will add to the existing literature on the epidemiology of HCV infection in prisoners including incident infection and risks. Findings will also evaluate the effectiveness of harm reduction services in Irish prisons and may inform future development of these services internationally.

## Methods

This study reports on the final part of a larger European HCV seek and treatment study (HepCare Europe project) and was a follow up to a previously reported prevalence study completed in 2017 [11, 19]. Ethical approval for the research was granted by The Mater Misericordiae University Hospital Research Ethics Committee (Ref:1/378/1839) and the Irish Prison Service (IPS) Ethics Review Committee.

### Study design

The original study included 422 participants of which 403 had HCV serology completed. This study found that 311 prisoners were HCV antibody negative and 92 (22.8%) were HCV antibody positive. A review of medical records found of those who were HCV antibody positive 23 (25%) had spontaneously cleared, 12 (13%) had a sustained virological response (SVR) and 53 (57%) had chronic infection.

**Fig. 1:**
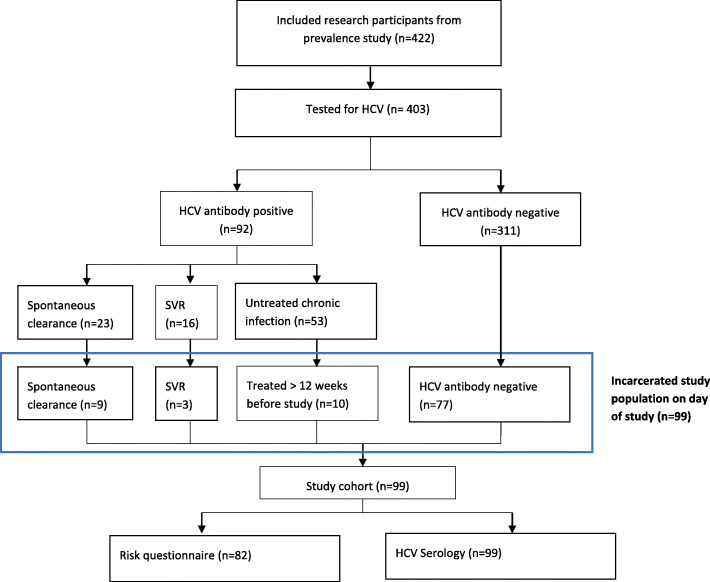
Incarcerated study population on day of study with serological evaluation at 18 months. HCV: Hepatitis C virus; SVR: Sustained virologic response

A review of prison and medical records found that 99 of the original study participants who were either; HCV antibody negative (*n* = 77), had achieved spontaneous clearance (*n* = 9) or had an SVR post-treatment (*n* = 13) were incarcerated at the time of this updated study. All 99 prisoners agreed to participate in this follow up cohort study and 82 agreed to complete a research-administered questionnaire which answers are presented in Table 2 (Fig. 1).

### Setting

Mountjoy Prison is a large urban prison which at capacity houses 538 sentenced male prisoners. The mean age of prisoners incarcerated at this location is 34 years, with a third serving sentences of less than 12 months and almost half on restricted regimes (protection prisoners) [4].

### Data collection

Data on study variables were collected from two sources; prisoners’ electronic medical records and a researcher-administered questionnaire. All prisoners routinely complete a nurse committal interview on the day of incarceration which is recorded in the prisoners’ medical records in the Prisoner Healthcare Management System (PHMS). From the medical records review, we collected the following variables: age, country of origin, incarceration history, pre-incarceration accommodation, drug and alcohol use and treatment, previous HCV-related risk behaviour (history of IDU, history of sharing syringes and drug-taking paraphernalia (non-injection drug use equipment such as pipes, spoons, etc.) history of tattooing and the sharing of toothbrushes and razors while incarcerated).

The risk questionnaire was developed and piloted by the research team in conjunction with national experts in the area of HCV infection and prisoner groups. The questionnaire covered the 18 month time period since the previous study and included questions on: incarceration history, history of current HCV-related risk behaviour both in the community and in prison (IDU, sharing syringes and other drug taking paraphernalia, receiving an unsterile tattoo) and engagement with harm reduction services (MAT and NSPs).

Questionnaires were completed prior to screening, but priority was given to completing screening over questionnaires, particularly in areas of the prison that housed enhanced-security prisoners. All study participants were given a patient information sheet and asked to sign a consent form. No inducements were offered.

### Serological testing

All participants were offered repeat serological screening for HIV, HBV, HCV antibody and reflex HCV RNA testing. First-line serological screening for hepatitis B virus (HBV), HCV and human immunodeficiency virus (HIV) was carried out using chemiluminescent microparticle immunoassays on the Architect i4000sr automated platform (Abbott, Chicago, United States). Confirmatory testing of reactive samples was carried out using alternative assays. HIV serological diagnosis was based on the Architect HIV Ag/Ab Combo (Abbott, Chicago, United States) and confirmatory testing for reactive samples using the VIDAS HIV DUO Ultra (BioMérieux, Marcy-l’Étoile, France) and HIV INNO-LIA HIV I/II Score (Innogenetics, Ghent, Belgium) assays. The Abbott Architect assays were used for HBV-associated markers and to characterise the HBV infection status. Serological screening for HCV included the anti-HCV test (Abbott Architect), a third-generation immunoassay and the Abbott Architect HCV Ag assay. All anti-HCV reactive samples negative for HCV antigen were further investigated to confirm the presence of anti-HCV antibodies using the anti-HCV VIDAS (BioMérieux) and INNO-LIA HCV Score (Innogenetics) assays. When HCV was confirmed serologically, molecular detection of HCV RNA was performed using the Abbott RealTi*me* HCV assay. HCV genotyping was conducted on samples with detectable HCV RNA using the Abbott RealTi*me* HCV Genotype II assay.

### Data analysis

All data were anonymised and coded, double-entered and checked. Statistical analysis was performed using the Statistical Package for Social Sciences (version 23.0; SPSS UK Ltd.; Chersey, United Kingdom). Data were assessed for normality and where necessary, data were log-transformed for normalisation purposes. Data in tables are primarily expressed as means with standard deviation (SD) or numbers with percentages.

## Results

### Demographic data

A total of 99 prisoners with a mean age of 32.2 years (yrs.) consented to participate in the study. 94% reported Ireland as their country of origin and 21% were homeless prior to incarceration. This cohort were first incarcerated in their late teens (mean age = 18.4 yrs.), had experienced multiple incarcerations (mean = 6.4) and had spent the majority of their young adult lives in prison (mean = 9.9 yrs.). Just over half (51%) of participants had a history of drug use, 43.9% a history of heroin use and 39% a history of IDU. The mean age of first drug use was 14.8 yrs., first heroin use was 18.8 yrs., and first IDU was 20.6 yrs.

In terms of historical risk factors for HCV acquisition, 42.7% gave a history of sharing drug taking equipment (paraphernalia), 17.3% of sharing syringes in the community, 22.5% of having had a prison tattoo and 23.8% a non-sterile community tattoo. Small numbers reported sharing a razor or toothbrush in a prison setting (2.5 and 1.3% respectively). A total of 42.1% reported having a history of methadone treatment, and the mean length of time on treatment was 6.3 years (Table 1).

**Table 1:** Patient demographics (medical records) on day of incarceration

Variable	Participants			
	Total	n	%	Mean (SD)
Age (years)	99			33.2 (9.1)
18–24		13	13.1	
25–34		46	46.5	
≥35		40	40.4	
Age at first incarceration (years)	81			18.4(5.9)
Episodes of incarceration	80			6.4 (7.6)
Total time incarcerated (years)	75			9.9 (6.8)
Age at first drug use (years)	66			14.8 (2.9)
Age at first heroin use (years)	41			18.8 (4.9)
Age at first injecting drug use (years)	66			20.6 (5.7)
Previous drug use (yes)	98	50	51	
Visible injection site (yes)	98	3	3.1	
Shared needles (yes)	98	5	5.1	
**Place of origin**	99			
Ireland		93	94	
Western Europe		1	1	
Eastern Europe		4	4	
Africa		1	1	
**Accommodation before incarceration**	71			
Secure		56	78.9	
Homeless		15	21.1	
**Risk factors for HCV acquisition**				
History of heroin use	82	36	43.9	
History of injecting drug use	82	32	39	
Shared needles in the community	75	13	17.3	
Shared drug-taking equipment in the community	75	32	42.7	
Shared razor in prison	80	2	2.5	
Shared toothbrush in prison	80	1	1.3	
Prison tattoo	80	18	22.5	
Unsterile community tattoo	80	19	23.8	
**Alcohol use**				
Alcohol problem before incarceration	81	12	14.8	
Treatment for alcohol use	55	3	5.5	
**Methadone maintenance treatment**				
History of methadone treatment	76	32	42.1	
Length of time on methadone maintenance treatment	20			6.3 (5.6)

*HCV* Hepatitis c virus

### Recent risk for HCV infection

Just under a quarter (23.5%: *n* = 99)) of the original study cohort were available to follow up (Fig. 1). Of these 82 completed the updated risk questionnaire on current risk factors for HCV acquisition (Table 2). 69 of this group (85.2%) remained incarcerated for the entire 18 month period since the previous study. The remainder (*n* = 13) had been released and re-incarcerated. Small numbers of patients engaged in recent IDU while incarcerated (*n* = 2) and on release into the community (*n* = 3). Only one patient reported sharing syringes in the community post incarceration. Sharing drug taking paraphernalia both in the community (*n =* 2) and in the prison (*n* = 4) was a little more common than needle sharing. 20% (*n* = 16) of participants were on MMT at the time of the study (Table 2).

**Table 2:** Results of updated risk questionnaire

Yes to variables	Risk Question
	Yes n (%)
Incarcerated since previous HCV test (*n =* 82)	69 (85.2)
Moved prison location (*n =* 82)	18 (22.0)
Engaged in IDU in prison (*n =* 82)	2 (2.4)
Engaged in community IDU while on release (*n =* 13)	3 (23.1)
Sharing needles in Prison (*n =* 82)	0
Sharing needles in the community while on release (*n =* 13)	1 (7.1)
Sharing drug paraphernalia in prison (*n =* 82)	4 (4.9)
Sharing drug paraphernalia in the community while on release (*n =* 13)	2 (15.4)
Unsterile tattoo in Prison (*n =* 82)	2 (2.4)
Unsterile tattoo in the community while on release (*n =* 13)	1 (7.1)
Methadone (*n =* 82)	16 (19.5)

*HCV* Hepatitis C Virus, *IDU* Injecting drug use

### Blood-borne virus serology

All 99 participants had repeat serology. The prevalence of HIV and HBV infection was 3% and HCV antibody infection was 22.2%. No new infections were identified either in those who had never been infected (*n* = 77), had self-cleared (*n* = 9) or achieved SVR (*n* = 12). There was one treatment failure (previously identified) (Table 3).

**Table 3:** Serology results

**Serology (*n =*** **99)**	**Number n (%)**
HIV antibody positive	3 (3.0%)
HBV antibody positive	3 (3.0%)
HCV antibody positive	22 (22.2%)
HCV antibody negative	77 (77.8%)
HCV RNA negative/self-clearance	9 (9.0%)
HCV RNA negative (SVR)	12 (12.1%)
HCV RNA positive	1 (1.0%) (treatment failure)

*HIV* Human immunodeficiency Virus, *HBV*, Hepatitis B Virus, *HCV* Hepatitis C Virus, *RNA* Ribonucleic Acid, *SVR* sustained viral response

## Discussion

Similar to other studies, this study found that drug users and PWID are over-represented in prisons [2, 3].. Over half of study participants had a history of drug use from an early age (14.5 yrs.). Heroin use and IDU were also common. Repeated incarcerations from a young age characterised this group. These findings reflect the recidivist nature of drug using prisoners and the limits of criminalisation and imprisonment in managing the health and social consequences of drug use and addiction. Of interest is the large turnover of prisoners in the 18 month period between the studies. Only 25% of the original cohort were available to retest. This reflects the dynamic nature of prison populations. Most prisoners serve short sentences (< 12 months) and are often transferred between prison locations during their prison sentence [20].

This study found no new HCV infections in a cohort of 99 prisoners who were followed up 18 months after having tested negative for HCV or having achieved SVR. This finding is welcome given the previously identified HCV-related risks linked with incarceration both nationally and internationally [3, 11, 13, 14, 21]. These risks include IDU, sharing syringes and other drug taking paraphernalia, having a non-sterile tattoo and factors independent of these but linked to incarceration such as sharing razors and tooth brushes and exposure to violent assaults [21, 22] Previous prison-based HCV incident studies have shown an in-prison incidence rate of 0.7–1.0 per 100-py in the overall prison population and 18–24/100py among prisoners with a history of injecting drug use [23, 24]. A 2013 systematic review and meta-analysis found a HCV incidence among general detainees of 1.4 per 100-py and 16.4 per 100-py in detainees with a history of IDU [3]. Studies reporting on HCV incidence in prisoners generally reported on larger numbers of prisoners and for longer follow up times than this study which may account for the higher incidence rates. However some studies have reported that prison is a protective factor for HCV infection due to reduced levels of IDU and access to MAT [25, 26]. This may also be the case in Irish prisons where MAT is easily accessible to all those needing treatment. This includes continuing prisoners on MAT while incarcerated and linking them to community MAT services on release [5, 27].

The low numbers of patients engaging in risk-behaviour reported in this study is encouraging, in particular in those who had achieved SVR. The risk of HCV re-infection among PWID following achieving an SVR is considered relatively low (1–5% per year), but there is considerable uncertainty around this estimate among those who continue to inject [28]. A number of studies have reported higher re-infection rates among current PWID and identified older age and IDU, at or post-treatment, as risk factors for re-infection [29]. The rate of HCV re-infection after successful treatment in prisoners is high, particularly among those who continue to inject drugs [30, 31]. Studies have reported an overall re-infection rate of 5.27 cases per 100-py. Re-infection was significantly higher among active drug users, HIV co-infected and those engaging in more than one risk behaviour after treatment [30, 31].

A small number (*n* = 13) of this study cohort had experienced prison release and re-incarceration between studies. Small numbers reported risk-behaviour which is encouraging. This finding may reflect the age of the cohort (mean age = 32.5 yrs.) and their existing knowledge of their HCV status. However the small numbers did not allow for comparison between those who remained incarcerated and those who had experienced release and re-incarceration. It also did not allow for comparisons between those never infected and those who had self-cleared or achieved SVR (previously infected). As already described most prisoners serve short sentences and many experience multiple cycles of release and re-incarceration. Community release can be particularly problematic and is associated with elevated risk of death and overdose [32–34]. People just released from prison have multiple issues to contend with and linking to healthcare is often not a priority. Prison release is also a stressful time which can trigger relapse and a return to high-risk behaviour [32, 35, 36]. Coordinating HCV treatment in these circumstances can also prove challenging. Supporting prisoners and creating robust links to MAT and other healthcare services immediately on prison release should be a key public health priority and an area for further research [27, 34].

The findings of this study support the ongoing need to develop and expand harm reduction services both in community and prison settings. Evidence shows that traditional harm reduction measures such as MAT and NSPs are effective in reducing self-reported syringe sharing [37]. Both interventions can reduce transmission of HIV and HCV, particularly when provided together. NSPs and MAT have been increasingly established but coverage remains poor and data on the quality of many of these services is unknown [38, 39]. These initiatives are fragile, politically unpopular, under-resourced and increasingly undermined by a ‘recovery agenda’ that prioritises abstinence. Although access to HCV screening and treatment for PWID seems to be poorer in prison than in the community, access to harm reduction measures is even more limited [39, 40]. Approximately 60 out of more than 10,000 prisons worldwide provide NSPs [39]. HCV prevention is almost exclusively limited to verbal advice, leaflets and other measures directed to cognitive behavioural change [40]. While the extent of multiple risk behaviours for HCV in prisons is challenging, the setting does offer an ideal opportunity to provide a range of evidence-based interventions that can reduce HCV infection [39]. These include MAT, NSPs and condom availability. These have the added advantage of reducing HIV transmission and, in the case of MAT, fatal overdose in the immediate post-release period. Despite the evidence-base for the effectiveness of these interventions in the reduction of the transmission of BBVs, there is poor coverage of these in prisons globally [41].

Incident studies in prison populations are rarely conducted. They are often difficult to design and implement. In this context the findings from this study are important since they add to what is a very small pool of data on incident HCV infection in prisoners. The findings are limited by the small numbers (*n* = 99) who were available for follow up and who completed the questionnaire (*n* = 82). The small numbers prevented any meaningful statistical comparison between those who remained incarcerated and those who had experienced released and re-incarcertaion and between those never infected and previously infected. A strength of the study is the use of serum samples rather than historical blood-borne virus screening data from chart records. The use of current serology is unusual in these types of studies and with high levels of both sensitivity and specificity for this method offers increased validity to this study over those using saliva samples. A further strength is that all eligible participants agreed to follow up serology.

The use of a research-completed questionnaire has both strengths and limitations. The researchers completing the questionnaire were unknown to the prisoners which may have allowed for more frank disclosure of risk behaviour. The design of our study allowed researchers to spend time with the prisoner and to add clarification regarding the meaning/interpretation of the questions. While overcoming the issue of literacy, it was time-consuming which impacted on completion rates. There are a number of further limitations to this study. It was single-site and only included male prisoners, making the findings more difficult to generalise both nationally and internationally. Data collected on the sharing of syringes and drug-taking paraphernalia did not distinguish between reception and distribution and did not report on the frequency of sharing. In addition, owing to the nature of the study we did have a small ‘n’ for a number of variables of interest, caution must be used in the interpretation of the results until they are replicated in larger studies. There were limits on allocated time to access prisoners, consequently, screening was prioritised over the completion of the risk questionnaire. This was particularly an issue with protection prisoners where enhanced security measures were in place.

## Conclusions

This study reported no new HCV infections over an 18 month period in a cohort of prisoners (*n* = 99) known to be not infected or successfully treated for HCV. Small numbers engaged in HCV-related risk behaviour while incarcerated and on release. A larger multi-site study is required to fully understand the rates and associated risks of HCV infection in Irish prisoners, but this preliminary study suggests that existing measures including MAT availability are protective in preventing HCV infection in this group.

## Data Availability

The datasets used and/or analysed during the current study are available from the corresponding author on reasonable request.

## Abbreviations

HCV: Hepatitis C virus
IDU: Injecting drug use
HIV: Human Immunodeficiency Virus
MAT: Medication assisted treatment
PWID: People who injects drugs
PY: Person-year
IPS: Irish Prison Service
NSPs: Needle syringe programmes
DAAs: Direct-acting antivirals
HBV: Hepatitis B virus
SVR: Sustained virologic response
PHMS: Prisoner Healthcare Management System
RNA: Ribonucleic acid
Yrs.: Years
SD: Standard deviation
